# Comorbidity of Obsessive-Compulsive Disorder and Schizophrenia in an Adolescent

**DOI:** 10.1155/2015/136835

**Published:** 2015-09-21

**Authors:** Ahmad Nabil Md. Rosli, Wan Salwina Wan Ismail

**Affiliations:** Department of Psychiatry, Universiti Kebangsaan Malaysia Medical Centre (UKMMC), Jalan Yaacob Latif, Bandar Tun Razak, Cheras, 56000 Kuala Lumpur, Malaysia

## Abstract

We report a case of a girl with a history of obsessive-compulsive disorder (OCD) subsequently exhibiting psychosis. She never attained remission since the outset. Initially she seemed to be resistant to most antipsychotics, namely, risperidone, haloperidol, paliperidone, quetiapine, and clozapine. However, she later responded remarkably better to risperidone after it was reintroduced for the second time. Recognizing and understanding the various pathogenesis of OCD or obsessive-compulsive symptoms (OCS) in schizophrenia are vital in laying out plan to manage the patient effectively.

## 1. Introduction

Obsessive-compulsive disorder (OCD) is considered as a distinct entity from schizophrenia as characterized in DSM 5 [1]. However cooccurrence is frequent. In this report we describe a case of childhood onset schizophrenia which was preceded by OCD. The challenges in managing psychosis and obsessive-compulsive symptoms (OCS) will be highlighted and the theory behind the occurrence of schizophrenia and obsession-compulsion will be discussed.

## 2. Case Report

She is a 17-year-old Chinese girl, the second of three siblings, who presented to the child psychiatry clinic at the age of 13 years, with prominent features of OCS. The symptoms started at the age of 11 years, during which she was obsessed with her body's physiological activities such as breathing and swallowing. In relation to that, she had compulsion to count her respiratory rate and how many times she had swallowed her saliva. She experienced intense feeling of distress when she resisted the repeated thoughts and behaviour. This occurred after her grandmother passed away and a year before her sixth-grade examination which was considered important in the Malaysian education system. However, the symptoms did not affect her social and academic functioning then. There was no history of psychiatric illness in her family.

Two years later, at the age of 13, the symptoms worsened. The obsessional theme changed from counting her breaths to thoughts of death and compulsion of cursing the dead, triggered by seeing the graveyards. As a consequence, she developed significant symptoms of depression including suicidal ideas. Later, she developed delusion of reference. Her social and academic functioning deteriorated and she was admitted to psychiatric ward for a month period. A diagnosis of severe OCD with comorbid major depressive disorder (MDD) with psychosis was made. She was treated with escitalopram 10 mg nocte and risperidone 0.5 mg bid. Sand tray therapy and cognitive behavioural therapy (CBT) were commenced later. The symptoms of OCD and MDD improved and she was discharged after a month of admission.

Despite good compliance to treatment, she never attained full remission. Subsequently, she developed psychotic symptoms characterized by delusions and disorganized thoughts and behaviour. It started with repeated doubts of the existence of God, ghost, and the holy spirits and the compulsion to get reassurance over her doubts. Later, the thoughts became delusional in nature, when she started to believe that God, ghost, and the holy spirits were watching over her all the time. She felt frightened and because of these beliefs she could not bathe on her own. She believed there was a CCTV placed in her house to spy on her. She could not go out from her home as she believed people were looking and talking about her. She never had mood or negative symptoms.

The diagnosis was revised to comorbid schizophrenia and OCD. The antipsychotic was changed from risperidone 0.5 mg bid to aripiprazole 5 mg daily in view of better prolactin profile and the fact that second-generation antipsychotic (SGA) like risperidone was understood to aggravate OCS in schizophrenia. Aripiprazole was gradually increased by 5 mg in two weeks up to 30 mg daily. However her delusion still persisted. Haloperidol 5 mg was then combined with aripiprazole for an adequate duration but the psychosis and OCS were still the same.

Since aripiprazole was already optimized, the next step taken was to substitute aripiprazole with quetiapine 50 mg while maintaining haloperidol at 5 mg. Quetiapine was increased to 100 mg a week later and then 200 mg subsequently. However she could not tolerate the medications due to extrapyramidal side-effect (EPSE), that is, tongue stiffness. Quetiapine and haloperidol were stopped and she was given trihexyphenidyl 2 mg bid for EPSE.

The next drug in line was olanzapine. There was an improvement in psychosis but it was not sustained for more than a month. Later the OCS worsened relative to psychosis despite adequate dose of 20 mg daily. Due to this, olanzapine was cross-tapered with paliperidone 3 mg. It was then gradually increased to 12 mg daily over the period of 4 months. Despite this trial, the result was still unsatisfactory. Thus, electroconvulsive therapy (ECT) was commenced while maintaining the paliperidone. However there was no improvement seen even after 12 ECT. Lexapro was increased to 30 mg daily over the period of two months. There was no full remission of OCS; however it managed to put her under control.

Although she had good adherence to medications plus adequate dosage and duration of treatment given, the psychotic symptoms appeared resistant as evidenced by the treatment failure of several antipsychotics. Depot injection of antipsychotic was also considered as an option but it was refused by patient and parents. Decision was then made to start clozapine. It was started at 12.5 mg daily and gradually increased up to 300 mg daily over the period of 2 months. In addition, she also developed tachycardia with a heartbeat ranging from 120 to 130 bpm, hypersalivation, and significant weight gain around 4 kg in 3 months without much improvement in her psychotic symptoms.

The team had finally decided to retry risperidone in view of limited options left for her. Furthermore, the dose of risperidone was not even optimized, only 0.5 mg bid during the first time. Risperidone was reintroduced at a lower dose of 0.5 mg bid and slowly increased by 0.5 mg in 2 weeks' time in view of previous history of EPSE. Interestingly, she responded fairly better when she was put back on it. At 2 mg daily, there were significant improvement in the OCS and positive symptoms; however she developed amenorrhea. There was mild delusion of reference but she was able to go out from the house and carried out her basic personal needs at home. The OCS was almost negligible that she started to think and plan about her future. Both of her parents had been very supportive of her throughout the time.

## 3. Discussion

OCD and schizophrenia commonly cooccur. About 25% of schizophrenia patients experienced OCS while, in 12%, criteria for diagnosis of OCD were fulfilled [2]. OCS in schizophrenia may manifest at 5 different junctures of time, that is, during prodromal period, prior to psychotic manifestation, at the onset of first psychosis, during the course of schizophrenia, and by antipsychotic induction [3]. As demonstrated in this case, the presence of OCD previously in one's life was associated with a greater risk of getting schizophrenia later on [4].

Comorbidity of OCD and schizophrenia has been explained by three main theories [5]. In the first theory, OCD was described as a prodrome of schizophrenia; OCD precedes schizophrenia but symptoms remit after psychosis emerged. OCD could also be the risk factor for schizophrenia where OCD precedes schizophrenia but symptoms persist [5].

Alternatively, they can also be understood if both are to be taken as having a common risk factor. A patient with schizophrenia may have vulnerability genes towards OCD and schizophrenia. The expression of OCD genes in a schizophrenia patient may be triggered by environmental factors at any stage of schizophrenia and hence varied its manifestation [6]. In this case the first onset of OCD was possibly triggered by psychological stress, that is, examination and death of grandmother. Subsequently, environmental factors which were common to both conditions might have expressed the schizophrenia genes.

**Figure 1:**
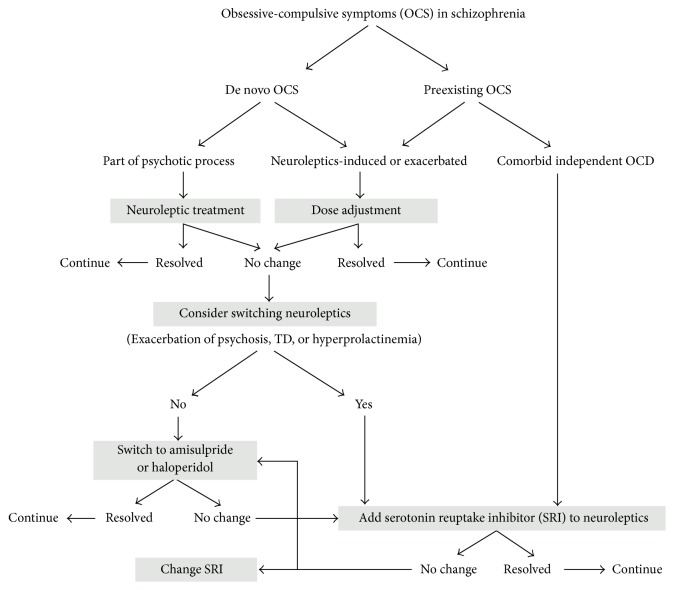
Algorithm of OCD/OCS management in schizophrenia. (Adapted from [7]. Adapted with permission.)

Treatment of OCD in schizophrenia should be individualized. The drug of choice to be used will depend on whether the OCS occurs de novo or preexists in relation to schizophrenia. SSRI can be added to an antipsychotic for patient with preexisting OCD while amisulpride is recommended for those who developed drug-induced OCS [7] (Figure 1).

In this case, olanzapine was noted to exacerbate the OCS and the child was treated with multiple antipsychotics and they were of adequate dosage and duration; however she was resistant to them. Kerwin and Bolonna (2005) suggested the reinstating of the best prior medication in a clozapine-resistant or intolerance patient [8]. From the history, risperidone was never fully optimized and with such suboptimal dose her symptoms were relatively alike in control as compared to other antipsychotics. Therefore risperidone was reintroduced and proven to be effective in controlling her psychosis.

## 4. Conclusion

This case highlights the challenges in managing this patient as well as the importance of recognizing the variability of obsessive-compulsive clinical presentation in the course of schizophrenia. Even though OCD that predates schizophrenia and persists independently thereafter tends to become treatment-resistant [7], it is not impossible for such a patient to achieve a considerable level of function and remission as exemplified in this case.
